# FPGA Implementation for 24.576-Gbit/s Optical PAM4 Signal Transmission with MLP-Based Digital Pre-Distortion

**DOI:** 10.3390/s24237872

**Published:** 2024-12-09

**Authors:** Sheng Hu, Tianqi Zheng, Chengzhen Bian, Xiongwei Yang, Xinda Sun, Zonghui Zhu, Yumeng Gou, Yuanxiao Meng, Jie Zhang, Jingtao Ge, Yichen Li, Kaihui Wang

**Affiliations:** State Key Laboratory of Integrated Chips and Systems, Fudan University, Shanghai 200433, China; 23210720170@m.fudan.edu.cn (S.H.); tqzheng23@m.fudan.edu.cn (T.Z.); 24110720124@m.fudan.edu.cn (C.B.); 22110720137@m.fudan.edu.cn (X.Y.); 22210720221@m.fudan.edu.cn (X.S.); 22210720335@m.fudan.edu.cn (Z.Z.); 22210720131@m.fudan.edu.cn (Y.G.); 22210720196@m.fudan.edu.cn (Y.M.); 23210720305@m.fudan.edu.cn (J.Z.); 23210720159@m.fudan.edu.cn (J.G.); 23210720197@m.fudan.edu.cn (Y.L.)

**Keywords:** digital pre-distortion, real-time communication, field-programmable gate array, pulse amplitude modulation 4-level, intensity modulation direct detection

## Abstract

In this work, we implemented a short-reach real-time optical communication system using MLP for pre-distortion. Lookup table (LUT) algorithms are commonly employed for pre-distortion in intensity modulation and direct detection (IM/DD) systems. However, storage limitations typically restrict the LUT pattern length to 9, limiting its effectiveness in compensating for nonlinear effects. A multilayer perceptron (MLP) can overcome this limitation by predicting errors and generating pre-distorted signals, thus replacing the extensive storage requirements of LUTs with minimal computational resources. The MLP-based digital pre-distortion (MLP-DPD) technique enables the creation of long-pattern LUTs for improved nonlinear compensation. In this work, an MLP-DPD scheme was implemented on a field-programmable gate array (FPGA). The FPGA was used to generate a 14.7456 GBaud pre-distorted pulse amplitude modulation 4-level (PAM4) signal. This signal was then transmitted over 20 km of standard single-mode fiber (SSMF). At the receiver, the parallel constant modulus algorithm (PCMA) was applied for signal processing. The bit error rate (BER) achieved met the 2.4 × 10^−2^ threshold for soft-decision forward error correction (SD-FEC), enabling a net transmission bit rate of 24.576 Gbit/s. This approach demonstrates the feasibility of using MLP-DPD for effective nonlinear compensation in high-speed optical communication systems with limited resources.

## 1. Introduction

With the rapid expansion of applications such as the metaverse, video streaming services, and the Internet of Things (IoT), the data traffic for data center interconnects (DCI) has surged, placing immense pressure on existing communication infrastructure. Due to its high bandwidth and low loss characteristics, optical fiber is well-suited to meet the demands of high-speed, large-volume data transmission, making short-reach optical communication an ideal solution for addressing this challenge [1,2,3,4,5,6]. Among various short-reach optical communication systems, intensity modulation and direct detection (IM/DD) technology has emerged as a mainstream choice due to its simple architecture, low cost, and ease of implementation [7,8,9]. IM/DD systems modulate the optical signal intensity directly and use a photodiode for direct signal detection, avoiding the need for complex coherent detection methods, which makes it highly suitable for short-reach, high-density connections. Furthermore, pulse amplitude modulation 4-level (PAM4) has become an effective method for meeting high bandwidth requirements, as it can transmit twice the amount of data within the same bandwidth, significantly improving the transmission efficiency of optical communication systems [10,11,12,13,14,15,16].

However, optoelectronic devices can introduce a range of nonlinear effects during high-power and long-distance transmission, such as self-phase modulation (SPM), cross-phase modulation (XPM), and four-wave mixing (FWM). These effects can cause signal distortion and degrade system performance. These nonlinear issues become more pronounced at high transmission speeds, requiring precise compensation techniques and optimized solutions [17,18,19]. Over the past decade, lookup table (LUT) digital pre-distortion (DPD) has been widely adopted in optical communication systems for its ability to effectively mitigate nonlinear effects [20,21]. However, the limited pattern length due to storage constraints restricts the performance of LUT-based approaches [22,23]. Researchers have also proposed DPD algorithms based on multilayer perceptron (MLP) and convolutional neural networks (CNN) [24,25].

Previous approaches have primarily been validated through offline experiments, often neglecting real-time implementation. Practical deployment, particularly in short-reach optical communication systems, requires consideration of hardware constraints such as limited computational capacity, power consumption, and cost. These stringent limitations make traditional LUT-based nonlinear compensation methods less feasible. The proposed MLP-DPD scheme addresses these challenges by providing an efficient solution that operates effectively within limited-resource environments without exceeding hardware limits. This makes MLP-DPD well-suited for real-time industrial applications, although further research and optimization are necessary to fully adapt these algorithms for widespread use.

Field-programmable gate arrays (FPGAs), with their real-time processing capability, high parallelism, and flexible programming features, are ideal hardware choices for meeting the demands of high-speed data transmission in optical communications [26,27,28,29,30,31].

This paper presents the first implementation of an MLP-DPD algorithm on an FPGA for a real-time short-reach IM/DD transmission system over a 20 km standard single-mode fiber (SSMF). The experiment successfully transmitted a 14.7456 GBaud PAM4 signal with a bit error rate (BER) below the SD-FEC threshold of 2.4 × 10^−2^, achieving a net data rate of 24.576 Gbit/s. At the transmitter, a 64-channel parallel MLP-DPD module was used for signal pre-processing, and its effectiveness in mitigating nonlinear effects was demonstrated by comparing the results with an unprocessed scenario. In addition, we discussed the impact of different tanh function implementations on the MLP prediction results. Based on the simulation results and resource constraints, we selected the PAA128-tanh scheme.

## 2. Principle

The basic principle of digital pre-distortion (DPD) is to compensate for distortion by subtracting the distortion value from the original transmitted signal. Although this principle is relatively simple, it is sufficiently practical and has a significant compensatory effect on nonlinearities in optical communication systems. Moreover, there are various implementation schemes.

The proposed method can be divided into four main parts: the traditional lookup table (LUT) pre-distortion scheme, the multilayer perceptron (MLP)-based pre-distortion scheme, fixed-point implementation, and the 64-channel parallel MLP-DPD. The first two parts describe the principles of LUT-DPD and MLP-DPD. The key difference between them lies in how the distortion value of the transmitted signal is obtained. LUT-DPD compensates by storing distortion values for different patterns, whereas MLP-DPD uses a trained MLP to predict the distortion values. The third part focuses on the fixed-point implementation and the realization of PAA-tanh, which plays an important role in the single-channel MLP-DPD implementation. The fourth part introduces the 64-channel parallel MLP-DPD, which successfully transforms the single-channel low-speed signal transmission architecture into a multi-channel high-speed transmission architecture.

### 2.1. Lookup Table (LUT)

In IM/DD systems, the electro-optical modulator, square-law detection in the receiver, and nonlinear effects induced by fiber transmission can impair the received signal, thereby limiting system performance. As a classical pre-distortion algorithm, the LUT method can reduce these nonlinear impairments and improve system performance.

**Figure 1:**
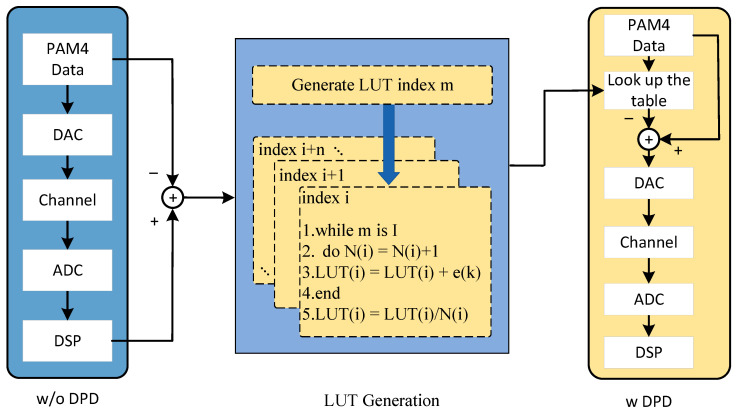
Principle of LUT-DPD.

Figure 1 illustrates the principle of the LUT. The pre-distortion technique is applied to a PAM4 short-reach IM/DD system to compensate for nonlinear distortions. The LUT-based digital pre-distortion (LUT-DPD) process consists of two stages—LUT generation and LUT utilization. The LUT is generated using training data. At the transmitter, a PAM4 training sequence $X_{train}(n)\in \{-3,-1,+1,+3\}$ of a certain length is given to a PAM4 short-reach IM/DD system to compensate for nonlinear distortions. The LUT-based digital pre-distortion (LUT-DPD) process consists of two stages—LUT generation and LUT utilization. The LUT is generated using training data. At the transmitter, a PAM4 training sequence $X_{train}(n)\in \{-3,-1,+1,+3\}$ of a certain length is transmitted, and the corresponding sequence $Y_{train}(n)$ is received and processed with digital signal processing (DSP) at the receiver. The error sequence e(n) is then calculated as the difference between the DSP-processed sequence and the training sequence using the following formula:

(1) $$e(n)=Y_{train}(n)-X_{train}(n),$$

Since nonlinear impairments are related to the transmitted sequence, the pre-distortion value for a symbol must consider the surrounding sequence within a certain length. Guided by this theory, the LUT is constructed as a table with 2 rows and $4^{M}$ columns. Initially, the first row of the LUT, which serves as the index, consists of all PAM4 sequences of length M, while the second row, representing the error values, is set to zero.

After obtaining the training sequence $X_{train}(n)$ and error sequence e(n), a sliding window of length M is applied to both sequences. At each step, a length M PAM4 sequence is extracted from $X_{train}(n)$ as the index i, and the central symbol e(k) is taken from e(n) for the following update:

(2) LUT(i)=LUT(i)+e(k),

(3) N(i)=N(i)+1,

Once the sliding window completes its traversal, the LUT is normalized for each index i using:

(4) $$LUT(i)=\frac{LUT(i)}{N(i)},$$

which completes the LUT generation. The LUT is then used for pre-compensation. For any given symbol X(k) in the transmitted sequence X(n), a sequence centered on X(k) with a total length of M, is selected as the index i. The following operation is then performed:

(5) $$X^{\prime }(k)=X(k)-LUT(i),$$

Here, $X^{\prime }(k)$ represents the pre-distorted signal produced by the LUT-DPD technique.

### 2.2. Multilayer Perceptron (MLP)-Based Digital Pre-Distortion (DPD)

The MLP is considered the most fundamental neural network model, consisting of multiple layers of neurons where the output of each layer is fully connected to the input of the next layer. The output of each layer h_i_ can be expressed as:

(6) $$h_{i}=f(W_{i}h_{i-1}+b_{i}),$$

where $W_{i}$ is the weight matrix of the *i*-th layer, $b_{i}$ is the bias of the *i*-th layer, and f(⋅) is the activation function. Common activation functions include ‘ReLU’, ‘Tanh’, and ‘Sigmoid’.

Since nonlinear distortion is related to the transmitted sequence, increasing the model length enhances the accuracy of characterizing the nonlinearity, leading to more precise pre-distortion. However, for LUT-based approaches, increasing the pattern length results in exponential growth in the training sequence length and storage requirements. Therefore, further improving LUT’s performance by increasing the model length is impractical. MLPs, with their simple structure and capability to express complex nonlinearities, offer a more resource-efficient approach by predicting the error directly rather than using a LUT to obtain the error for generating the pre-distorted signal.

**Figure 2:**
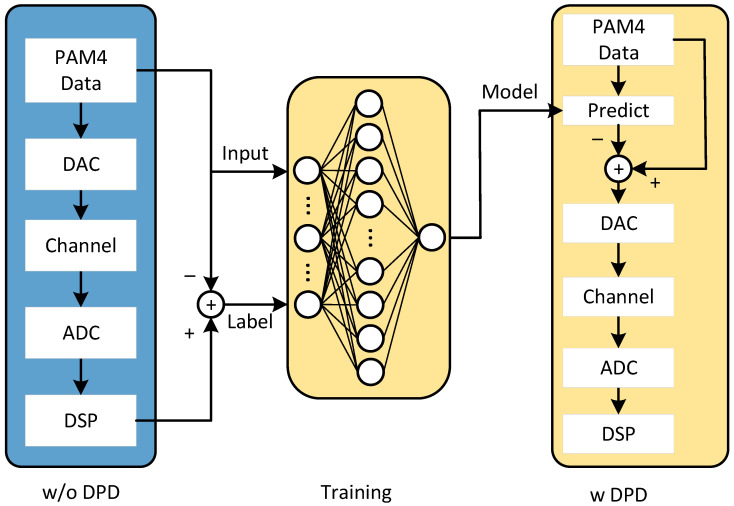
Principle of MLP-DPD.

The principle of MLP-DPD is illustrated in Figure 2. Comparable to the LUT and other neural networks, the algorithm consists of two stages: training the MLP and using the MLP-DPD. The process begins similarly to the LUT algorithm, where a training sequence $X_{train}(n)$ of a certain length is generated. After multiple transmissions via the DAC, channel transmission, ADC sampling, DSP processing at the receiver, and averaging, the sequence $Y_{train}(n)$ is obtained. The repeated transmissions and averaging at the receiver help mitigate the effects of additive Gaussian white noise (AWGN). According to Equation (1), the error e(k) is computed as the difference between $Y_{train}(n)$ and $X_{train}(n)$.

A sliding window is then applied to $X_{train}(n)$ and e(n), yielding multiple pairs of indices i of length M and error values e(k). The indices i serve as features, and the error values as labels serve for training the MLP. Based on the findings of Chen, the number of features at the input layer, i.e., the selected model length, has the most significant impact on performance, and using the ‘tanh’ activation function in the hidden layer provides better results than the commonly used ‘ReLU’. Since predicting the error based on the transmitted sequence pattern is a regression problem, the output layer activation function is set to ‘purelin’, and the mean squared error (MSE) is used as the loss function. Hyperparameters are continuously adjusted to ensure network convergence and optimal performance, with the final selected hyperparameters shown in Table 1.

After training the MLP, the trained model is used to predict the error based on the transmitted sequence pattern, and the signal is processed as follows:

(7) $$X^{\prime }(k)=X(k)-MLP_{output}(i),$$

where $X^{\prime }(k)$ represents the MLP-DPD signal.

### 2.3. Fixed-Point Quantization

For MLP-DPD, most of the computational effort is concentrated on training the MLP. Using the MLP to predict the error and generate the pre-distortion signal requires minimal computational resources and latency, which is a prerequisite for deploying MLP-DPD in real-time short-range IM/DD communication systems. The parameters of the MLP trained on a PC are represented as floating-point numbers; however, performing floating-point arithmetic on an FPGA consumes significantly more resources than fixed-point arithmetic. Therefore, converting the MLP parameters to a fixed-point format can save substantial resources.

The fixed-point format is defined as QL1.L2, where the total bit length of the fixed-point number is L1, and the fractional part length is L2. The QL1.L2 format can represent values in the range $\left(-2^{L1-L2-1},2^{L1-L2-1}\right]$. To quantize a parameter p within this range to QL1.L2 format, it is multiplied by a scaling factor and converted to a binary number of the given length using two’s complement. Specifically, the scaling is performed as follows:

(8) $$p_{trunc}=Round(p\cdot 2^{L2}),$$

where Round(⋅) represents the rounding function. The quantized value, $p_{trunc}$, is then converted into an L1-bit two’s complement binary number, yielding the QL1.L2 fixed-point representation, $p_{fixed}$, of parameter p.

The inputs, weights, and biases of the MLP can be quantized using the aforementioned approach. However, the activation function used in the MLP is tanh, which is defined as:

(9) $$\tanh(x)=\frac{e^{x}-e^{-x}}{e^{x}+e^{-x}}.$$

Calculating the tanh function involves exponentiation and division, both of which have unacceptably high complexity when implemented on an FPGA. To reduce resource consumption, a piecewise approximation approach (PAA) is used. The range is divided into multiple equal-length subintervals. The endpoints of each subinterval, represented as $\left(x_{n},y_{n}\right)$ and $\left(x_{n+1},y_{n+1}\right)$, are used to perform Lagrange interpolation, resulting in the following approximation:

(10) $$P(x)=y_{n}\cdot l_{n}(x)+y_{n+1}\cdot l_{n+1}(x),$$

(11) $$l_{n}(x)=\frac{x-x_{n+1}}{x_{n}-x_{n+1}},$$

(12) $$l_{n+1}(x)=\frac{x-x_{n}}{x_{n+1}-x_{n}}.$$

According to the above interpolation formula, within each subinterval, the tanh function is approximated using a linear function:

PAAtanh(*x*) = *w_n_* + *b_n_*, *x* ∈ subinterval *n*. (13)

**Figure 3:**
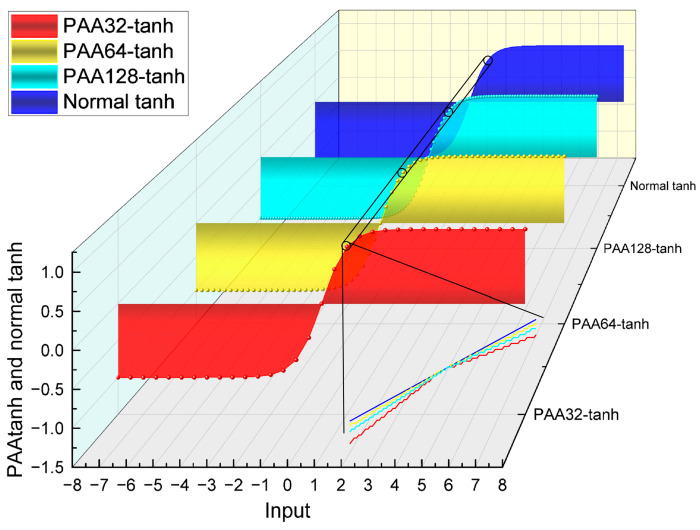
Plot of PAA-tanh versus the original tanh when divided into different numbers of segments.

**Figure 4:**
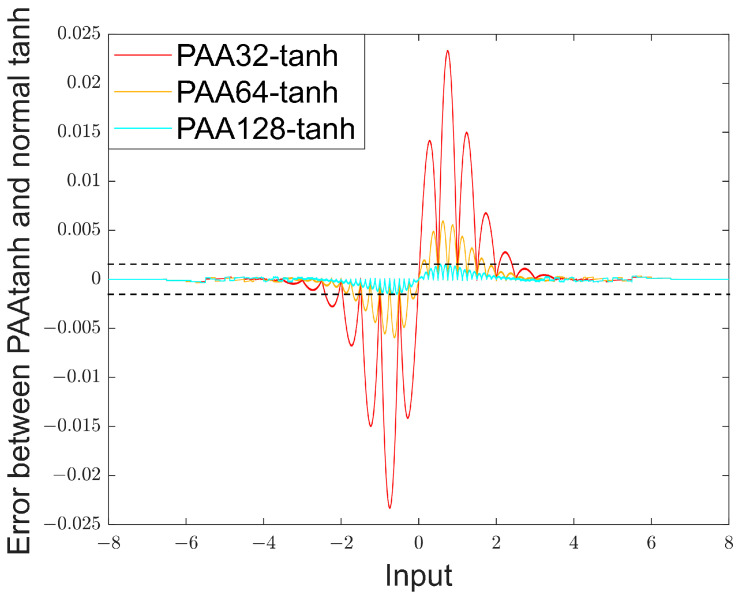
Comparison of the errors of the two when divided into different segments.

Figure 3 and Figure 4 show the difference between the normal tanh and the PAA-tanh. PAA32, PAA64, and PAA128 represent dividing the input range into 32, 64, and 128 subintervals, respectively. Since the error of PAA128-tanh is less than 1.6 × 10^−3^, and this accuracy exceeds the limitations of other system components, PAA128-tanh was chosen as the implementation of tanh to avoid unnecessary hardware resource expenditure.

The output of the hidden layer, $h_{1out}$, must still be multiplied by weight $w_{2}$ and added to bias $b_{2}$. Within each subinterval, these two linear operations can be combined to further reduce resource consumption, as expressed by the following formulas:

(14) $$w_{\tanh\&l2}=w_{2}\circ w_{PAA\tanh}(h_{1in}),$$

(15) $$b_{\tanh\&l2}=w_{2}\cdot b_{PAA\tanh}(h_{1in})+b_{2},$$

A∘B represents the Hadamard product of A and B, that is, the new matrix obtained by multiplying matrices of the same dimension element by element. In other words, their Hadamard product C=A∘B is defined as $c_{ij}=a_{ij}\cdot b_{ij}$. It is commutative, associative, and distributive over addition. It is widely used in deep learning, signal processing, and image processing for element-wise operations, such as feature extraction and filtering. The $w_{PAA\tanh}(h_{1in})$ and $b_{PAA\tanh}(h_{1in})$ represent the coefficients $w_{n}$ and biases $b_{n}$ of the PAAtanh(x) for each element in $h_{1in}$, corresponding to the subinterval n where the element is located. The final output $h_{2out}$ is then given by:

(16) $$h_{2out}=w_{\tanh\&l2}\cdot h_{1in}+b_{\tanh\&l2}.$$

This $h_{2out}$ represents the final output of the MLP and corresponds to the predicted error value.

### 2.4. 64-Channel Parallel MLP-DPD

It is important to note that the FPGA platform operates at a clock frequency of 230.4 MHz, which is significantly lower than the 14.7456 GBaud, the symbol rate of the MLP-DPD-PAM4 signal. To meet the required data throughput, a 64-channel parallel MLP-DPD module is necessary.

**Figure 5:**
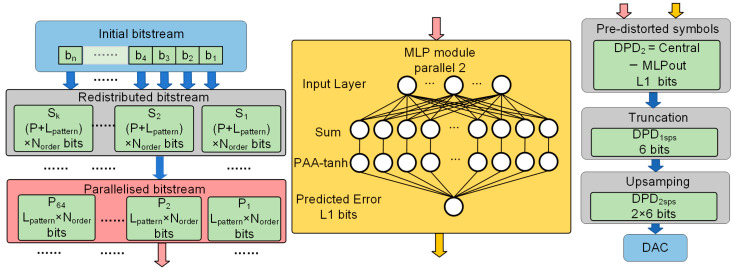
Principle of 64-channel parallel MLP-DPD.

Based on the principles of MLP-DPD, the fixed-point quantization scheme, and the parallelization strategy, we successfully deployed a 64-parallel MLP-DPD module on the FPGA development platform. Figure 5 illustrates the data flow diagram of the 64-channel parallel MLP-DPD module. The original pseudo-random binary sequence (PRBS) is divided into blocks of 128 bits, with each block concatenated with the adjacent $(L_{pattern}-1)\cdot N_{order}/2$ bits of data to form a reallocated sequence $S_{i}$. At each clock cycle, the reallocated sequence $S_{i}$ is parallelized, resulting in a 64-channel sequence $P_{i}$, where each channel $P_{i}$ consists of $L_{pattern}\cdot N_{order}$ bits. Each path $P_{i}$ is fed into a separate MLP-DPD module, where the forward propagation is performed sequentially through operations, including weighted multiplication, accumulation, bias addition, PAA-tanh, and further accumulation to predict the error for each pattern.

Simultaneously, the central symbol of each sequence $P_{i}$ is identified, and its bit-width is expanded. These predicted errors and the central symbols are then used to compute the pre-distorted signal. The pre-distorted signal is subsequently fed to the DAC for transmission.

## 3. Experiment Setup

**Figure 6:**
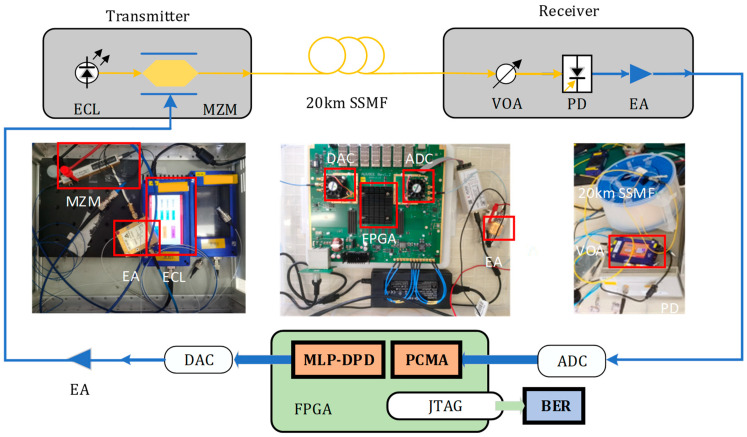
Experimental setup and hardware implementation.

To validate the effectiveness of NN-DPD in a real-time fiber optic system, we implemented a short-range fiber communication system based on the principles shown in Figure 6. We selected the Xilinx AUV901 FPGA development platform, which features a 6-bit 29.4912 GSa/s digital-to-analog converter (DAC) and a 6-bit 29.4912 GSa/s analog-to-digital converter (ADC) for the deployment of real-time PAM-4 signal pre-emphasis, transmission, reception, and equalization. It is important to note that the performances of the DAC and ADC are affected by the signal frequency. The DAC’s maximum output voltage is 1.38 V, while the ADC’s maximum input voltage is 0.78 V. For example, at a signal frequency of 15,000 MHz, the DAC’s spurious-free dynamic range (SFDR) is 28 dB, and at 10,000 MHz, the effective number of bits (ENOB) for the ADC is 4.7 bits.

A randomly generated bitstream, representing the pseudo-random binary sequence (PRBS) indicated by the read-only memory, is continuously fed into the transmitter’s pre-emphasis module. After pre-emphasis, the signal is truncated to 6 bits and upsampled by a factor of two to match the DAC’s resolution and update rate. Following these processing steps, the DAC converts the resulting 14.7456 GBaud 6-bit real-time MLP-DPD-PAM4 discrete signal into an analog electrical signal. This electrical signal is then amplified by a wideband electronic amplifier (SHF 100 BP) with a gain of 17 dB and subsequently converted to the optical domain by a Mach–Zehnder modulator (MZM). The optical carrier for the MZM is provided by an external cavity laser (ECL) with an output power of 14.5 dBm, a linewidth of less than 100 kHz, and a wavelength of 1550 nm. The modulated optical signal is coupled into a 20 km standard single-mode fiber (SSMF) and transmitted to the receiver.

At the receiver, the optical signal’s power is controlled by a variable optical attenuator (VOA) before being detected by a photodiode (PD) using square-law detection to convert it into an electrical signal. This signal is amplified by an electronic amplifier (EA) and then sampled by the ADC at a rate of 29.4912 GSa/s. The sampled signal from the ADC is sent to the Xilinx AUV901 FPGA. Since the received signal is sampled twice per symbol, the onboard PCMA module can be used for equalization without the need for additional resampling. Using Xilinx’s Integrated Logic Analyzer (ILA), the ADC-captured signal and the signal after PCMA equalization are transmitted to a personal computer (PC) via the Joint Test Action Group (JTAG) interface for bit error rate (BER) analysis.

## 4. Experimental Results and Discussions

### 4.1. FPGA Implementation of MLP-DPD

Based on the MLP-DPD model and the parameter fixed-point quantization scheme, with a 64-channel parallel implementation, the MLP-DPD can be deployed on the Xilinx AUV901 FPGA development platform. For the data sequence, the real-time MLP-DPD module on the FPGA performed pre-distortion with a fixed-point scheme. The various resources consumed by the MLP-DPD module are shown in Table 2.

### 4.2. Transmission of OB2B and 20 km SSMF

**Figure 7:**
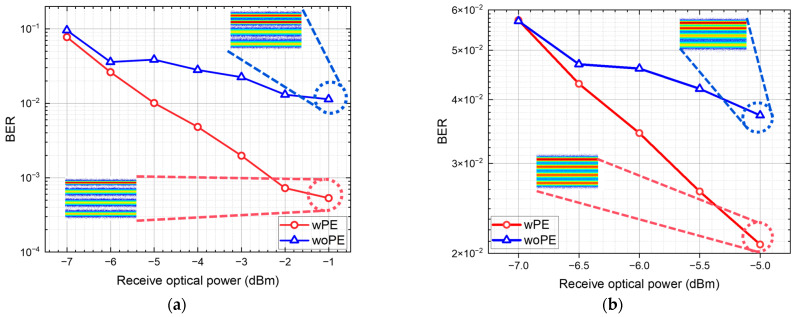
Experimental result graph and heatmap of received PAM4 signal. (**a**) Relationship between the BER curve and received optical power under BTB conditions. (**b**) BER curve after 20 km SSMF transmission.

After selecting the simulation parameters and schemes, real-time experiments were conducted to verify the effectiveness of MLP-DPD in mitigating the nonlinear effects on PAM4 signals in IM/DD systems. The initial experiments focused on the optical back-to-back (B2B) configuration. Figure 7 shows the bit error rate (BER) performance curves as a function of received optical power. In Figure 7, the blue curve represents the scheme without pre-distortion, while the red curve represents the scheme using MLP for pre-distortion. For each received optical power level, both PCMA as well as MLP-DPD and PCMA processing were applied. The improvement in nonlinear effects was assessed based on the BER heatmaps of the received signals. The lower the BER, the more evenly the four signal levels are represented in the heatmap, indicating that the nonlinear effects of the signal have been more effectively compensated.

The experimental results demonstrate a significant enhancement in system performance with MLP-DPD, showing a noticeable reduction in BER. In the range of −3 dBm to −1 dBm received power, the BER meets the 3.8 × 10^−3^ HD-FEC standard. At −1 dBm, the MLP-DPD and PCMA scheme achieved the best performance, with the signal after PCMA equalization exhibiting a more uniform and clearer four-level structure compared to the results without MLP-DPD, resulting in a substantial reduction in BER. As the received power increases, nonlinear effects become more pronounced, and since the MLP was trained with data at −1 dBm, significant performance improvements are observed at this power level. The performance at other power levels is still better than without pre-distortion, indicating a certain degree of robustness for MLP-DPD.

Subsequently, we investigated the performance of the PCMA as well as the MLP-DPD and PCMA schemes for PAM4 signals after transmission over a 20 km standard single-mode fiber (SSMF). Figure 7b presents the BER versus received optical power curves after 20 km SSMF transmission. Due to fiber attenuation, the maximum received optical power is reduced by approximately 4 dB. The results show that the trend of the curves remains consistent with the B2B transmission results. The MLP-DPD module consistently improves system performance, exhibiting effective BER reduction, even under strong nonlinear effects. At −5 dBm, the MLP-DPD and PCMA scheme again demonstrated optimal performance. The PAM4 signal, after PCMA equalization with MLP-DPD, exhibited a more uniform heatmap with four distinct levels. Figure 7 also illustrates the heatmaps and probability density distributions of the PAM4 signal for the two schemes. The PAM4 signal with MLP-DPD exhibits more uniform spacing between levels, indicating a certain suppression of the nonlinear effects.

Both in the B2B system and the 20 km SSMF system, the reduction in BER and the more uniform lines in the heatmap demonstrate the effectiveness of the MLP-DPD algorithm in compensating for the system’s nonlinear effects.

### 4.3. System Time Stability

**Figure 8:**
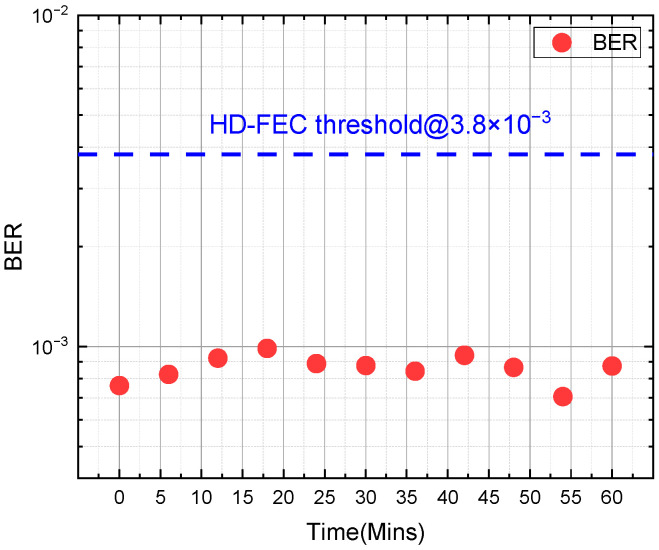
Real-time BER measurement within one hour (ROP is −1 dBm).

To verify the temporal stability of the system, we conducted an hour-long optical B2B experiment, collecting data at 6 min intervals. Figure 8 shows the BER performance over time, with small fluctuations in BER between 7 × 10^−3^ and 9 × 10^−3^ over the one-hour time scale, all satisfying the HD-FEC threshold. The transmit optical power is kept at −1 dBm.

## 5. Conclusions

In summary, we implemented an FPGA-based real-time IM/DD system capable of supporting a 29.4912 Gbit/s PAM4 signal transmission over a 20 km SSMF, achieving the SD-FEC threshold of 2.4 × 10^−2^. Considering an overhead of 20%, the net bit rate is 24.576 Gbit/s. The MLP was used to enhance the digital pre-distortion (DPD) traditionally performed by the LUT, incorporating techniques such as fixed-point quantization, piecewise approximation, and computation merging to reduce computational complexity and hardware resource usage. This allowed for a 64-way parallel deployment on the FPGA.

Compared to the traditional LUT algorithm, the MLP-DPD enables longer pattern lengths, conserves resources, lowers power consumption, and more effectively mitigates nonlinear effects. The successful validation of this module suggests that it can be integrated into transmitter modules in the future to achieve better system performance and higher capacity.

## Figures and Tables

**Table 1:** Hyperparameters of the MLP. FC layer means fully connected layer.

Hyperparameter	Option
Network architecture	Input layer, 35 features
	FC layer, 11 output
	Activation, ‘tanh’
	Output layer, 1 output
	Activation, ‘purelin’
Optimizer	Adam

**Table 2:** Resources consumed by the MLP-DPD module.

Resource	Amount
CLB LUTs	212,869
CLB Registers	515,650
CARRY8	37,312
F7 Muxes	16,000
Block RAM	7.5
DSPs	704

## Data Availability

The raw/processed data required to reproduce these findings cannot be shared at this time as the data also form part of an ongoing study.
